# Umbilical cord miRNAs to predict neonatal early onset sepsis

**DOI:** 10.1371/journal.pone.0249548

**Published:** 2021-05-07

**Authors:** Linda M. Ernst, Leena B. Mithal, Karen Mestan, Vivien Wang, Kathy A. Mangold, Alexa Freedman, Sanchita Das

**Affiliations:** 1 Department of Pathology and Laboratory Medicine, NorthShore University HealthSystem, Evanston, IL, United States of America; 2 Department of Pathology, University of Chicago Pritzker School of Medicine, Chicago, IL, United States of America; 3 Division of Infectious Diseases, Department of Pediatrics, Northwestern University Feinberg School of Medicine, Ann & Robert H. Lurie Children’s Hospital of Chicago, Chicago, IL, United States of America; 4 Division of Neonatology, Department of Pediatrics, Northwestern University Feinberg School of Medicine, Ann & Robert H. Lurie Children’s Hospital of Chicago, Chicago, IL, United States of America; 5 Institute for Policy Research, Northwestern University, Evanston, IL, United States of America; 6 Department of Obstetrics and Gynecology, NorthShore University HealthSystem, Evanston, IL, United States of America; University of Wisconsin - Madison, School of Veterinary Medicine, UNITED STATES

## Abstract

**Objective:**

To determine if miRNA (miR) expression in umbilical cord blood and umbilical cord tissue differs between neonates with early onset sepsis (EOS) versus neonates without true infection.

**Methods:**

Retrospective case-control study design of human patients with EOS (n = 8), presumed sepsis (N = 12) and non-infected control patients (N = 21). Differential expression of >300 miRs was examined using the MIHS-3001ZE-miScript miRNA PCR Array Human miFinder 384HC. Expression levels of miRs were normalized using the global Ct mean of expressed miR and compared between groups. Data analysis was performed using GeneGlobe data analysis software. Ratios of over and under-expressed miRs were calculated and compared between groups using receiver operating characteristic (ROC) curves.

**Results:**

Both umbilical cord plasma and umbilical cord tissue revealed several miRs with differential expression with little overlap between the two specimen types. The most overexpressed miR in plasma of EOS patients was miR-211-5p and the most overexpressed in EOS cord tissue was miR-223-5p. ROC curves comparing the ratios of over and under-expressed miRs for EOS patients and controls resulted in an area under the curve of 0.787 for cord plasma (miR-211-5p/miR-142-3p) and 0.988 for umbilical cord tissue (miR-223-5p/miR-22-3p), indicating good discrimination.

**Conclusions:**

miRs show differential expression in EOS versus non-infected controls and presumed sepsis. A ratio of over and under-expressed miRs can provide a potentially sensitive and specific diagnostic test for EOS.

## Introduction

While acute chorioamnionitis is a relatively common condition affecting 1–4% of all births in the United States [1], infection of the neonate is less common affecting only 0.77–1 per 1000 livebirths [2, 3]. However, neonatal sepsis continues to be a cause of significant morbidity and mortality, particularly in preterm infants. It is the third most common cause of neonatal deaths, and unfortunately, despite advances in prenatal care and antibiotic prophylaxis, early onset sepsis (EOS) remains a leading cause of infant morbidity and mortality responsible for almost 0.5 million infant deaths annually [4]. A major gap in addressing EOS is the lack of reliability in identifying those infants who are infected amongst the many infants born with a history of clinical chorioamnionitis. Blood culture remains the current gold standard for diagnosis of EOS despite question of its low sensitivity in neonates and delayed diagnosis [5]. Therefore, many premature neonates are treated with broad-spectrum antibiotics [6] which can lead to serious adverse consequences including disrupted gastrointestinal colonization, necrotizing enterocolitis, invasive fungal infections, and development of antimicrobial resistance [7]. To address this gap, a more reliable method for diagnosis of EOS is needed which utilizes either neonatal blood or tissue and identifies evidence of the fetal/neonatal inflammatory response to infection. Previous work has illustrated acute phase reactants [5] and even bacterial 16SrRNA DNA [8] can be found in the cord blood of patients with EOS; however, other blood and tissue markers such as miRNAs (miRs) have only recently been explored in EOS [9–12].

miRs are small (20–23 nucleotides), non-coding RNA molecules involved in post-transcriptional fine tuning of eukaryotic gene expression by targeting specific messenger RNAs (mRNA) and leading to either degradation of the target mRNA or suppression of translation of the target mRNA [13]. Therefore, miRNAs are key factors in the regulation of inflammatory mediators that eventually determine the immune response to infection. In addition, pathogen-induced signaling by toll-like receptors (TLRs), which is important in recognition of microbes and initiation of the host immune response, has been shown to either regulate and/or be regulated by miRNAs [13]. Therefore, we hypothesized that miR expression patterns in umbilical cord blood and/or umbilical cord tissue may help to distinguish neonates with EOS from those without true infection, and ultimately lead to more accurate assessment of EOS in neonates. Our aim was to test this hypothesis in a cohort of neonates for whom EOS status was well-characterized and compare their miR expression profiles with neonates with presumed sepsis and with non-infected controls.

## Methods

### Patient/specimen selection

We employed a retrospective case-control study design of human patients with EOS (defined by positive blood culture in the first 72 hours of life), neonates with clinical signs and symptoms of sepsis (respiratory distress, hemodynamic instability/poor perfusion, temperature instability, hypoglycemia, elevated immature to total neutrophil ratio, low absolute neutrophil count, or elevated C-reactive protein) who received antibiotics for at least 7 days with negative cultures (referred to as “presumed sepsis”) and non-infected control patients. Frozen cord blood plasma, collected at time of delivery, processed, and archived according to previously published methods [14], and formalin fixed paraffin embedded (FFPE) umbilical cord tissue specimens were selected from an ongoing longitudinal cohort study conducted at Northwestern Prentice Women’s Hospital in Chicago, IL, United States of America. Eligible patients for the parent study were live births ranging from 23–36 weeks of gestation with available cord blood. Infants with prenatal diagnosis of congenital anomalies were excluded. For this study, a subset of mother-infant dyads was selected from an existing cohort with complete clinical, placental, and culture data. Maternal and infant clinical data were obtained using abstraction from the Northwestern Electronic Data Warehouse which included data on prenatal care, intrapartum management, pregnancy complications, and birth outcomes. Banked umbilical cord blood plasma and FFPE umbilical cord tissue was obtained from 41 neonates: 8 with EOS, 12 with presumed sepsis, and 21 non-infected controls. The study was approved by the Northwestern University Feinberg School of Medicine Institutional Review Board (IRB protocol # STU00022673). Parental written informed consent was obtained for each patient prior to enrollment. Subsequent investigation was conducted according to the principles expressed in the Declaration of Helsinki.

### Extraction of RNA and miRNA array

Total RNA including miRNA was extracted from plasma samples using the miRNeasy Serum/Plasma Advanced Kit (Cat. No. 217204, Qiagen, Valencia, CA), following the manufacturer’s protocol. Briefly, 200 μl plasma was mixed with 60 μl lysis buffer and incubated at room temperature for 3 min. A spike-in control (3.5 μl of *Caenorhabditis elegans* miRNA 39 cel-miR-39-3p) was added at this stage to control for extraction efficacy and sample variability. This was followed by further incubation to ensure precipitation of protein. The supernatant was then passed through a spin column and RNA eluted. For RNA extraction from tissue samples, each FFPE umbilical cord tissue block was cut into four 10 μm thickness sections. The sliced tissues were deparaffinized, and the total RNAs were extracted using the miRNeasy FFPE Kit (Qiagen, Valencia, CA) according to the manufacturer’s instructions. The procedure included lysing the tissue with proteinase K and heat treatment, followed by a DNase treatment before passing through a spin column and elution of the RNA.

Reverse transcription polymerase chain reaction (RT-PCR) amplification was performed using the miScript II RT Kit (Cat No. 218161, Qiagen, Valencia, CA) following the manufacturer’s instructions. Briefly, 20 μl reaction mixture was made of 2 μl miScript reverse transcriptase mix, 4 μl miScript HiSpec buffer, 2 μl miScript nucleic acids mix, and RNase-free water and RNA template. The reactions were carried out with incubation for 60 min at 37°C for reverse transcription, following which the reaction was heated for 5 min at 95°C to inactivate miScript Reverse Transcriptase Mix on a thermal cycler (Applied Biosystems, Waltham, MA).

MIHS-3001ZE-miScript miRNA PCR Array Human miFinder 384HC (Cat No. 331223, Qiagen, Valencia, CA; https://b2b.qiagen.com/~/media/genetable/mi/mm/30/mimm-3001ze) was used for real-time PCR profiling of mature miRNA, which contains 372 well-annotated miRNAs and positive and negative controls. Interested researchers will be able to access/utilize the proprietary PCR analysis, owned by Qiagen, in the same way as the authors in this study. The preparation was performed according to the manufacturer’s instructions. Briefly, the arrays were performed using the miScript SYBR Green PCR Kit (Cat No. 218076, Qiagen, Valencia, CA), which contains the miScript Universal Primer (reverse primer) and QuantiTect SYBR Green PCR Master Mix. After addition of each cDNA template, the mixtures were added to each well in 10 μl reaction volumes. The PCR plate was then spun down and loaded onto the Quant Studio 12K (Thermo Fisher Scientific, Waltham, MA). The run was programmed according to the manufacturer’s instructions and data was acquired using the QuantStudio software.

### Placental pathology

Placental pathology reports were obtained and reviewed for each patient. Maternal and fetal acute inflammatory response in the membranes, chorionic plate, and umbilical cord was staged (stage 1–3) according to Amsterdam criteria [15]. Maternal acute inflammatory response consisted of acute subchorionitis, chorionitis and amnionitis with or without necrosis. Fetal acute inflammatory response consisted of acute chorionic vasculitis, acute umbilical phlebitis, acute umbilical arteritis, funisitis and necrotizing funisitis. Findings such as the presence of bacteria on the extraplacental membranes or umbilical cord seen on either the hematoxylin & eosin stains or Brown & Hopps tissue Gram stain were recorded. The presence of acute villitis or intervillositis, acute inflammatory cells infiltrating the chorionic villi or intervillous space, was also noted. Other placental characteristics such as the presence of chronic inflammation in the membranes, basal or chorionic plate, villi or intervillous space, findings within the spectrum of maternal vascular malperfusion (MVM), such as decidual vasculopathy, infarction, retroplacental hematoma, accelerated villous maturation or distal villous hypoplasia, and fetal vascular malperfusion (FVM), such as fetal vascular thrombi and avascular villi, were also recorded using Amsterdam criteria [15].

### Data analyses

Expression levels of miRNAs were normalized using the global Ct mean of expressed miRNA and compared between groups. Data analysis was performed using GeneGlobe data analysis software (https://www.qiagen.com/us/shop/genes-and-pathways/data-analysis-center-overview-page/?akamai-feo=off) provided by Qiagen. Differentially expressed miRNAs were identified by the dCt method. Fold change and regulation differences in the expression were calculated for each miRNA between the EOS, presumed sepsis and control group. P values were determined using student’s t-test for each miRNA. The most over-expressed and under-expressed miRNA in the EOS group compared with control group were then used to calculate ratios which were compared between the three study groups. Ratios were log-transformed to satisfy normality assumptions. Differences in the three groups were determined using ANOVA and Student’s t-test. Receiver operating characteristic (ROC) curves were also generated to evaluate the performance of the ratios in distinguishing cases from controls. Because preterm controls are inherently from abnormal pregnancies, a principal component analysis (PCA) was conducted to investigate whether miRNA expression patterns clustered by primary reason for preterm delivery, either preterm labor or preeclampsia.

Differences between the three study groups for demographic and placental pathology data were calculated using student’s t-test and ANOVA for continuous variables and Fisher’s exact test for categorical variables. P values <0.05 were considered statistically significant. Analyses were conducted using IBM SPSS Statistics for Windows, Version 25.0 (IBM Corp., Armonk, NY). ROC curves were generated using the pROC package and PCA was conducted using prcomp in R 3.6.1 (R Core Team, 2019).

## Results

### Patient/specimen selection

Demographic and clinical characteristics of the study groups are showed in Table 1. There were no statistically significant differences noted between the study groups with reference to gestational age, birthweight, infant gender, or preterm premature rupture of membranes. Patients in both the EOS group and presumed sepsis group had a higher prevalence of maternal clinical chorioamnionitis than controls. There was no significant difference between the prevalence of maternal clinical chorioamnionitis in the EOS group and presumed sepsis group.

**Table 1:** Demographic and clinical characteristics of the study groups.

	Control	Presumed	EOS	P
	N = 21	N = 12	N = 8	
**Gestational age, weeks, mean ± SD**	31.0 ± 2.4	30.3 ± 2.4	30.9 ± 3.1	0.87
**Birthweight, Grams, mean ± SD**	1699 ± 622	1469 ± 457	1683 ± 780	0.58
**Infant gender, n (%)**				
** Male**	9 (43)	9 (75)	5 (63)	0.22
** Female**	12 (57)	3 (25)	3 (37)	
**Multiple gestation**				
** No**	14 (67)	8 (67)	6 (75)	0.99
** Yes**	7 (33)	4 (33)	2 (25)	
**PPROM**				
** No**	14 (67)	7 (58)	3 (63)	0.41
** Yes**	7 (33)	5 (42)	5 (37)	
**Maternal**				
**Preeclampsia**				
** No**	16 (76)	10 (83)	8 (100)	0.47
** Yes**	5 (24)	2 (17)	0 (0)	
**Maternal**				
**Chorioamnionitis**				
** No**	21 (100)	9 (75)	5 (63)	<0.01
** Yes**	0 (0)	3 (25)	3 (37)	
**Placental inflammation** *				
**Maternal acute inflammation**				
** No**	11 (52)	6 (50)	0 (0)	0.05
** Yes**	10 (48)	6 (50)	8 (100)	
**Fetal acute inflammation**				
** No**	18 (86)	7 (58)	0 (0)	<0.01
** Yes**	3 (14)	5 (42)	8 (100)	

Within the EOS group (N = 8), four neonates grew *Escherichia coli* in the blood, two patients grew Group B Streptococcus (GBS), one patient grew *Listeria monocytogenes*, and one patient grew *Haemophilus influenza*. As part of the study design, neonates in the presumed sepsis group and control group had no positive cultures during their hospitalization and none developed late onset sepsis.

### Placental pathology

Maternal and/or fetal acute inflammation in the placenta was present in all 8 of the EOS cases. While maternal acute inflammation was seen more commonly in the EOS group than the presumed sepsis or control group, there was not a statistically significant difference between the groups. There was a statistically significant difference between the prevalence of fetal acute inflammation between controls (14%), presumed sepsis (42%) and EOS (100%), P<0.001. See Table 1. Among the EOS patients with fetal acute inflammation, 7/8 had stage 2 inflammation with neutrophils present in both the umbilical vein and artery and the presence of funisitis with neutrophils in Wharton’s jelly. No patients showed necrotizing funisitis. Bacterial organisms and acute villitis were present in one EOS case with positive blood culture for *Escherichia coli*. Inflamed fetal chorionic vessels with thrombosis were also seen in another EOS case with positive blood culture for *Escherichia coli*. No other placental pathology was significantly different between the study groups.

### miRNA expression in cord blood plasma

One control cord blood plasma sample was excluded because of inadequate volume of plasma for extraction. Extraction of miRNA and analysis of cord blood plasma samples was technically successful in all of the remaining 40 samples. Six miRs were differentially expressed in EOS vs controls with a P value <0.01 and fold change >2 (see Table 2). The most upregulated miR in plasma of EOS versus controls was miR-211-5p and the most downregulated miRNA was miR-142-3p. Four miRs were differentially expressed in the plasma of presumed sepsis versus controls, and none of the miRs overlapped with those that were differentially expressed in EOS vs controls. Expression of two miRs, miR150-5p and miR206 was significantly downregulated in EOS vs presumed sepsis.

**Table 2:** Top miRNA differences between groups–cord blood plasma.

**Cord Blood Plasma**		
**EOS vs Control**		
**miRNA Name**	**Fold Change**	**p value**
hsa-miR-211-5p	5.42	0.000788
hsa-miR-331-5p	3.16	0.002431
hsa-miR-181d-5p	-2.05	0.006363
hsa-miR-146b-5p	-2.66	0.007861
hsa-miR-142-3p	-2.7	0.008193
hsa-miR-193a-5p	2.57	0.009197
**Presumed Sepsis vs Control**		
**miRNA Name**	**Fold Change**	**p value**
hsa-miR-532-5p	-2.4	0.001462
hsa-miR-363-3p	-2.65	0.001797
hsa-miR-15a-5p	-2.41	0.004855
hsa-miR-202-3p	2.45	0.007692
**EOS vs Presumed Sepsis**		
**miRNA Name**	**Fold Change**	**p value**
hsa-miR-206	-28.18	0.006982
hsa-miR-150-5p	-3.85	0.00961

**Fig 1:**
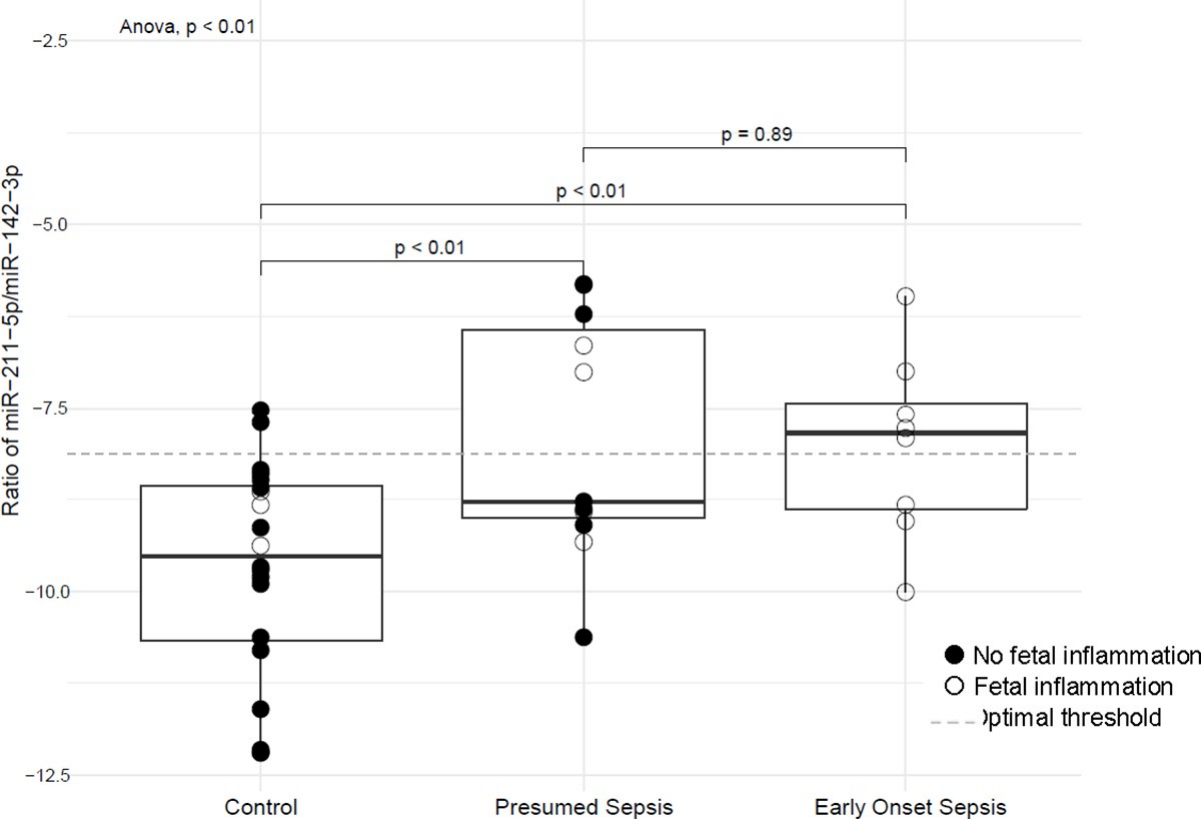
Ratio of miR-211-5p/miR-142-3p in umbilical cord blood plasma. This graph depicts the median, interquartile range, and outliers for the three study groups: non-infected controls, presumed sepsis, and EOS. The ratio is significantly higher in EOS than non-infected controls and presumed sepsis.

Using the raw Ct values in the umbilical cord plasma normalized to SNORD96A [16], the ratio of miR-211-5p, the most overexpressed miRNA in EOS vs controls, to miR-142-3p, the under-expressed miRNA in EOS vs controls and presumed sepsis, was calculated. The ratios ranged from 0.00000507 to 0.00299963 (log-transformed range: -12.19 to -5.81) and the mean value of the EOS group was statistically significantly different from the control group (P<0.01) but not the presumed sepsis group (P = 0.89). See Fig 1.

### FFPE umbilical cord tissue

Two FFPE umbilical cord samples were excluded from analysis because there was inadequate tissue in the block or inadequate placental pathology information (one from the control and one from the presumed sepsis group). Extraction of miRNA and analysis on the remaining 39 FFPE umbilical cord tissue samples was technically successful in all samples. Fifteen miRs were differentially expressed in EOS vs controls with a P value <0.01 and fold change >2 (see Table 3). The majority of the miRs were overexpressed in EOS compared to controls while only one miR was under-expressed with fold change >2, miR-22-3p (fold change -2.01, p = 0.001328). The highest significant fold changes in EOS were seen with miR-223-5p and miR-223-3p which both demonstrated a >14-fold increase over controls. Two miRs that were overexpressed in EOS were also overexpressed in patients with presumed sepsis: miR-626 and miR-363-5p (see Table 3). In addition, there were seven overexpressed miRs that were uniquely overexpressed in the presumed sepsis group compared to controls (see Table 3). The most overexpressed miR in the presumed sepsis group was miR-661 with a fold change of 3.41 and this miR also had the lowest P-value, P = 0.000891. Two miRs were differentially overexpressed in EOS vs presumed sepsis (miR-338-3p and miR146b-5p). Interestingly, both of these miRs were also highly overexpressed in EOS versus controls.

**Table 3:** Top miRNA differences between groups–FFPE umbilical cord.

**FFPE Umbilical Cord Tissue**		
**EOS vs Control**		
**miRNA Name**	**Fold Change**	**p value**
hsa-miR-338-3p**	2.37	0.000013
hsa-miR-132-3p	2.2	0.000172
hsa-miR-155-5p	2.21	0.001162
has-miR-22-3p	-2.01	0.001328
hsa-miR-132-5p	2.17	0.00143
hsa-miR-223-5p	18.48	0.001999
hsa-miR-142-5p	3.76	0.002675
hsa-miR-626*	2.33	0.00432
hsa-miR-223-3p	14.77	0.004686
hsa-miR-9-5p	2.73	0.005326
hsa-miR-155-3p	2.69	0.00651
hsa-miR-146b-5p**	2.4	0.007039
has-miR-186-3p	2.09	0.00807
hsa-miR-182-3p	2.08	0.00869
hsa-miR-363-5p*	2.27	0.009506
**Presumed Sepsis vs Control**		
**miRNA Name**	**Fold Change**	**p value**
hsa-miR-661	3.41	0.000891
hsa-miR-92a-1-5p	2.09	0.001451
hsa-miR-122-5p	2.42	0.001983
hsa-miR-363-5p*	2.21	0.003907
hsa-miR-122-3p	2.21	0.004996
hsa-miR-626*	2.01	0.005202
cel-miR-39-3p	2.22	0.006075
hsa-miR-218-1-3p	2.08	0.008125
hsa-miR-106a-3p	2.23	0.008538
**EOS vs Presumed Sepsis**		
**miRNA Name**	**Fold Change**	**p value**
hsa-miR-338-3p**	2.7	0.000944
hsa-miR-146b-5p**	2.5	0.006552

*miRs expressed in EOS vs control and Presumed sepsis vs control

** miR over-expressed in EOS vs control and Presumed sepsis

**Fig 2:**
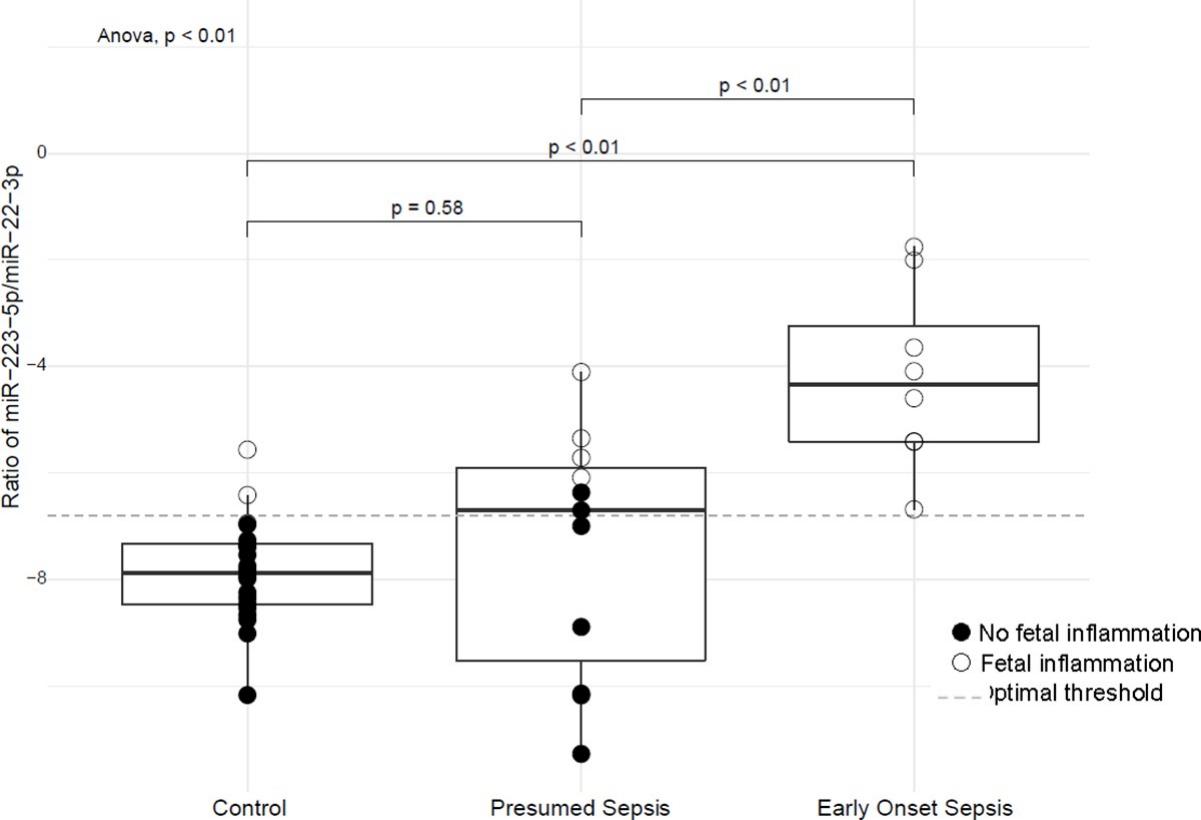
Ratio of miR-223-5p/miR22-3p in FFPE umbilical cord tissue. This graph depicts the median, interquartile range, and outliers for the three study groups: non-infected controls, presumed sepsis, and EOS. The ratio is significantly higher in EOS than non-infected controls. The ratio is intermediate in presumed sepsis.

Using the raw Ct values in the FFPE umbilical cord tissue normalized to SNORD96A, the ratio of miR-223-5p, the most overexpressed miRNA in EOS vs controls, to miR-22-3p, the under-expressed miRNA in EOS vs controls, was calculated. The ratios ranged from 0.00001278 to 0.175312512 (log-transformed range: -11.27 to -1.74) and the mean value for the EOS group was statistically significantly different from the control group (P<0.01) and the presumed sepsis group (P<0.01). See Fig 2.

**Fig 3:**
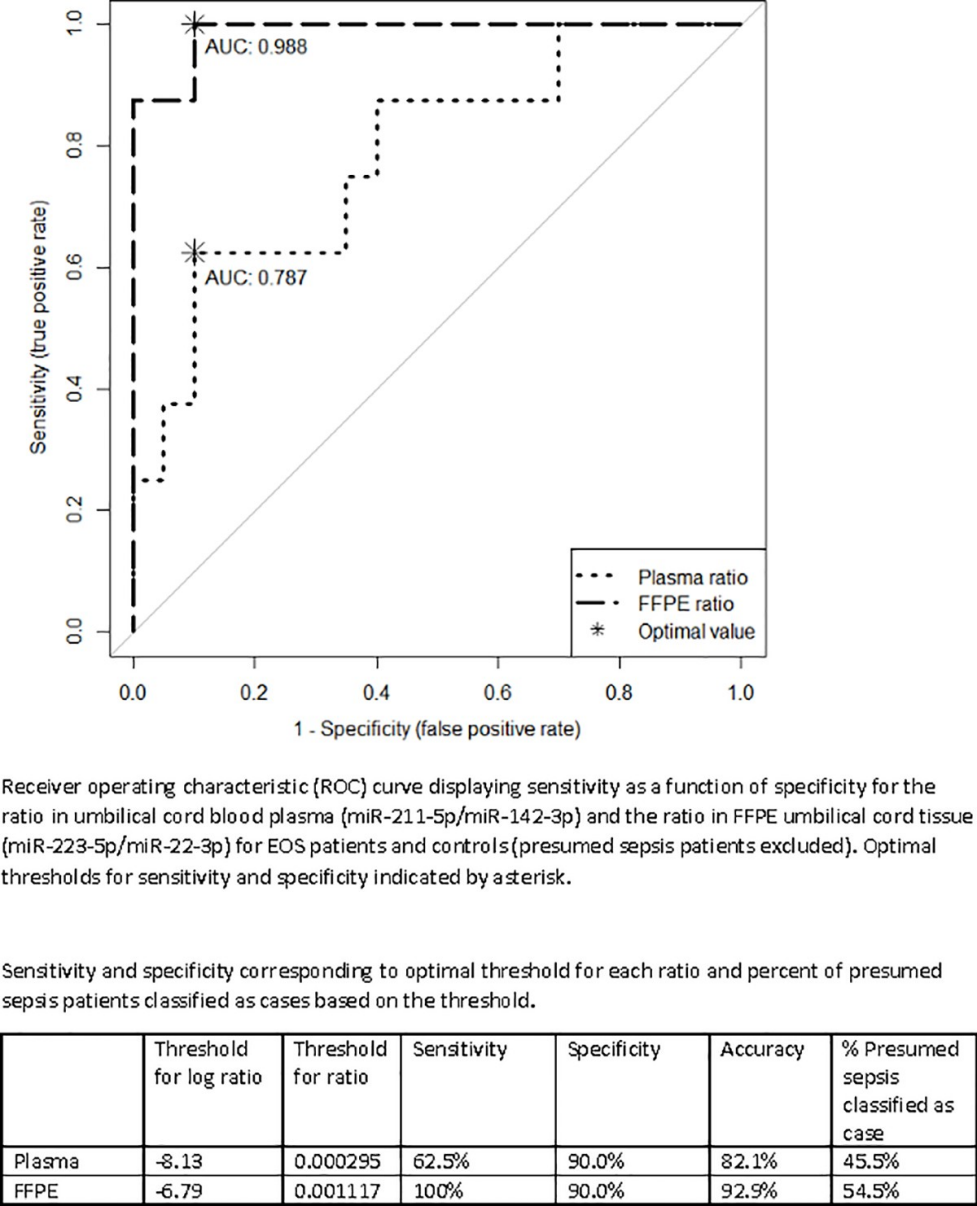
Receiver operating characteristic (ROC) curve displaying sensitivity (true positive rate) as a function of 1 –specificity (false positive rate) for ratio in cord blood plasma (miR-211-5p/miR-142-3p) and the ratio in FFPE cord tissue (miR-223-5p/miR-22-3p) for EOS patients and controls (presumed sepsis patients excluded).

ROC curves comparing the EOS group to controls for both ratios are shown in Fig 3. The area under the curve (AUC), a measure of accuracy, was 0.787 for the cord blood plasma ratio and 0.988 for the FFPE cord tissue ratio. This indicates that the ratios, especially the FFPE ratio, are good measures to distinguish EOS patients from controls [17].

**Fig 4:**
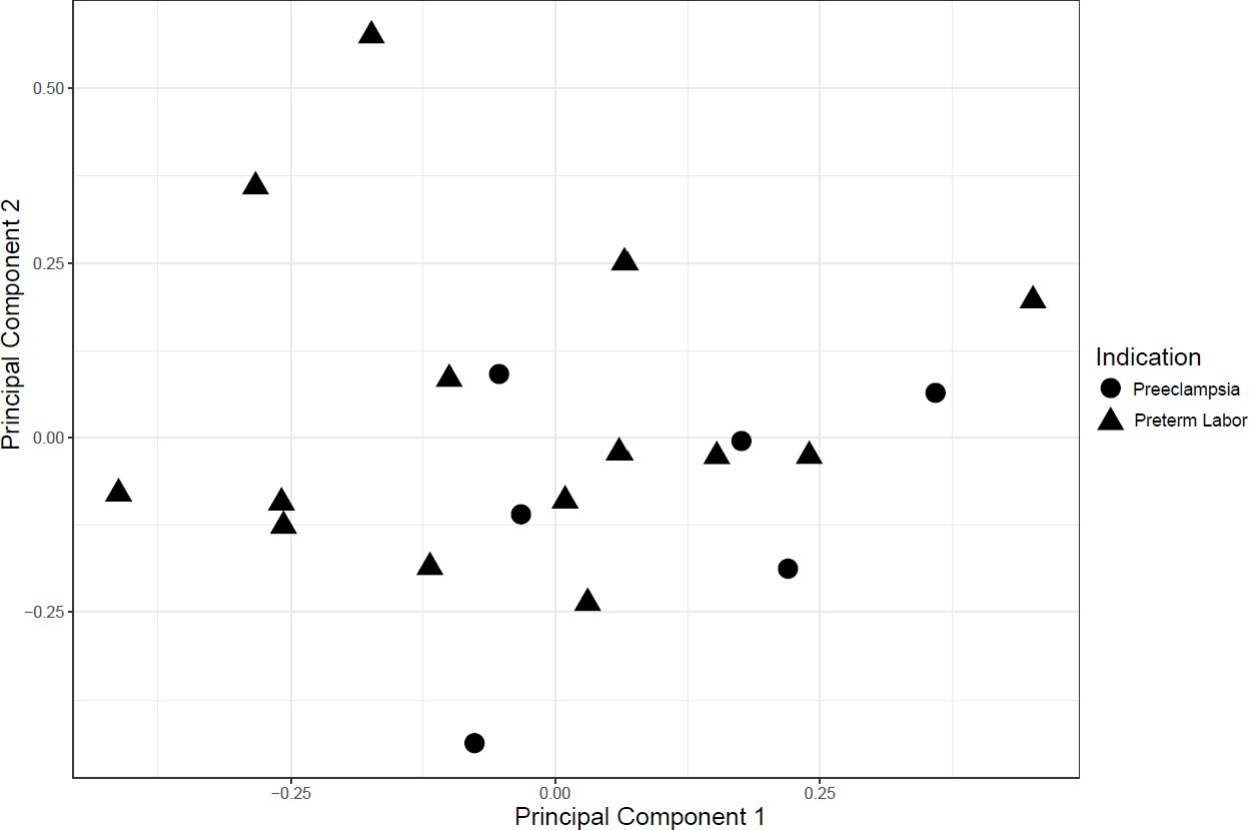
Plot of principal components 1 and 2 for miRNA expression in FFPE umbilical cord tissue of controls. Shapes indicate primary reason for preterm delivery.

The PCA analysis of miRNA expression in FFPE among preterm controls did not reveal clustering by primary reason for preterm delivery (Fig 4).

## Discussion

Our data show that miRNA expression in umbilical cord blood plasma and umbilical cord tissue differs between EOS and non-infected controls. The most significantly overexpressed miR in umbilical cord blood plasma in EOS was miR-211-5p. The role of miR-211-5p has not been well-studied in sepsis, but it has been implicated as a regulator of a variety of cellular processing including angiogenesis [18], maintenance of joint homeostasis [19], and autophagy-dependent apoptosis [20]. miR-211 has recently been proposed to play a role in sepsis modulation [21] as there is some evidence that miR-211 levels drop after exposure to LPS in cell culture [22] and that miR-211 could protect against inflammation in osteoarthritis [23]. In our study, miR-211 was upregulated in umbilical cord blood plasma, but not in the umbilical cord tissue. The significance of this discordance is not entirely clear.

The most significantly overexpressed miR in umbilical cord tissue in EOS was miR-223 with miR-223-5p showing a >18-fold increase in EOS vs controls and miR-223-3p also showing a >14-fold increase in EOS vs controls. Neither miR223-5p nor miR223-3p distinguished patients with presumed sepsis from controls or patients with EOS from presumed sepsis. miR-223 was not significantly up or downregulated in the plasma of patients with EOS.

miR-223 is a highly conserved miRNA involved in the regulation of hematopoiesis, particularly myeloid cell differentiation and function, with numerous downstream targets including STAT3, STAT5, NF-KB, and Hsp90 [9, 24]. It is expressed in myeloid cells and upregulated during granulocytic differentiation. Since the pathophysiology of EOS is typically an ascending bacterial infection which leads to granulocytic mobilization from the umbilical cord vessels as part of the fetal inflammatory response, our finding of upregulation of miR-223 in umbilical cord tissue and not in the plasma of EOS patients seems plausible. miR-223 has also been reported by other investigators to be upregulated in the plasma of pediatric patients with sepsis [25]. However, other investigators have shown a downregulation of miR-223 in EOS [9] or no significant difference between sepsis and controls [10, 26] when studying plasma or serum levels. Our findings, therefore, suggest that the miR-223 level may be more predictive of EOS when tested from the umbilical cord tissue. Interestingly, umbilical cord tissue is easily obtained at the time of birth without need for performance of an invasive procedure such as collection of blood on a neonate. Further study is needed to determine if our findings in FFPE umbilical cord tissue can be reproduced in fresh umbilical tissue acquired at the time of birth as this represents an easily obtainable specimen with potential important diagnostic information.

To our knowledge, no previous studies have examined umbilical cord tissue miRs in the setting of EOS and compared the results with umbilical cord blood plasma miRs, as we have done in this study. Interestingly, but not unexpectedly, the miRs that were differentially expressed in cord blood and cord tissue were not identical. In fact, there was essentially no significant overlap between the miRNA profiles. Few studies have compared levels of miRs in tissue with circulating miRs and looked for discordance. One such study reported a positive correlation between tissue and circulating miRNAs in patients with colorectal carcinoma [27]. Another study of gastric carcinoma reported some differences between the miRs upregulated and downregulated in tissues versus plasma [28]. Since miRs are so vital to the exquisite regulation of cellular process at the local level, it stands to reason there would be some discordance between tissue miRs and circulating miRs, as seen in our data.

If laboratory methods interrogating miRs to predict neonatal infection are to be successful, the simple detection of an important miR is not likely to be adequate. However, the use of ratios of overexpressed miRs/under-expressed miR could add to the specificity and sensitivity. This technique has been used in other clinical settings such as pancreatic carcinoma [29, 30] to improve diagnostic accuracy. In our study, using miR ratios, we were able to significantly separate EOS patients from non-infected controls. Both ratios also demonstrated good discrimination between EOS patients and presumed sepsis patients. In particular, in umbilical cord tissue, we were able to achieve a sensitivity of 100% and a specificity of 90% using a threshold established from the normalized ratio of miR-223-5p/miR-22-3p to detect EOS. When we applied this threshold to the presumed sepsis group, it identified 6 of 11 as EOS cases. While this is a small sample and remains exploratory, our results suggest that some patients treated empirically for sepsis in the presumed sepsis group have miR profiles more similar to non-infected controls while some have miR profiles more similar to EOS. Therefore, the culture negative sepsis may represent a molecularly distinct group of patients. The ability to separate out molecular profiles associated with the lack of infection versus potential infection in this presumed sepsis group is particularly important as a highly specific test for EOS could allow neonatologists to confidently discontinue antibiotics and avoid prolonged antibiotic exposure, perhaps even prior to receipt of a negative blood culture result. Further investigation of this group is needed to determine who is truly infected, perhaps by more sensitive molecular techniques, because currently with a negative blood culture there is no “gold standard” to guide therapy.

There are several limitations to our study. First, our results are based on a small number of EOS and presumed sepsis cases. Second, our analysis compared preterm EOS and presumed sepsis cases to preterm controls without sepsis, and our results may not generalize to term births. Additionally, preterm controls may have different indications for preterm delivery than preterm EOS and presumed sepsis cases, including differences in preterm labor, preterm rupture of membranes, and elective C-section without labor due to preeclampsia. These factors could explain any differences observed in miRNA expression profiles. However, a PCA analysis of miRNA expression profiles did not reveal distinct clustering by preeclampsia or preterm labor, two common reasons for preterm delivery. Lack of clustering suggests that the underlying reason for preterm delivery may not explain the observed differences in miRNA expression across the EOS cases, presumed sepsis cases, and controls. Finally, presumed sepsis cases were identified based on clinical characteristics, which include abnormal inflammatory markers. Similarities in miRNA expression between EOS and presumed sepsis cases may be due to similar clinical profiles, which may not be specific to neonatal infection.

A sensitive and specific, rapid diagnostic test to distinguish true neonatal infection from a less serious exposure in utero to infected amnionic fluid has immense potential to reduce morbidity and cost of unnecessary antibiotic therapy and could contribute to the success of antimicrobial stewardship programs. Our data suggest that miRs may be an important part of the diagnostic work-up for EOS, and may help separate truly infected neonates from uninfected patients. Our work suggests that the establishment of cut-offs for miR ratios could be incorporated into clinical practice to provide an objective measurement of likelihood of infection and has the potential to become the cornerstone of early diagnosis, appropriate clinical management and antibiotic stewardship.

## Supporting information

S1 File Demographic and placenta infl data. (XLSX)

S2 File FFPE raw Ct counts. (XLS)

S3 File Plasma raw Ct counts. (XLSX)
